# Concerted action of IFN-α and IFN-λ induces local NK cell immunity and halts cancer growth

**DOI:** 10.18632/oncotarget.10272

**Published:** 2016-06-24

**Authors:** Ahmed Lasfar, Andrew de la Torre, Walid Abushahba, Karine A Cohen-Solal, Ismael Castaneda, Yao Yuan, Kenneth Reuhl, Andrew Zloza, Elizabeth Raveche, Debra L Laskin, Sergei V Kotenko

**Affiliations:** ^1^ Department of Pharmacology and Toxicology, Ernest Mario School of Pharmacy, Rutgers, the State University of New Jersey, Piscataway, NJ, USA; ^2^ Rutgers Cancer Institute of New Jersey, New Brunswick, NJ, USA; ^3^ Department of Surgery, New Jersey Medical School, Rutgers, The State University of New Jersey, Newark, NJ, USA; ^4^ St Joseph's Medical Center, Paterson, NJ, USA; ^5^ Department of Microbiology, Biochemistry and Molecular Genetics, Center for Immunity and Inflammation, University Hospital Cancer Center, New Jersey Medical School, Rutgers, the State University of New Jersey, Newark, NJ, USA; ^6^ Section of Surgical Oncology Research, Department of Surgery, Rutgers Robert Wood Johnson Medical School, Rutgers, The State University of New Jersey, New Brunswick, NJ, USA; ^7^ Department of Pathology, New Jersey Medical School, Rutgers, The State University of New Jersey, Newark, NJ, USA

**Keywords:** hepatocellular carcinoma, HCV, IFN therapy, IFN-α/IFN-λ combination, tumor immunity

## Abstract

Hepatocellular carcinoma (HCC) is the most prevalent type of liver cancer. No significant improvement has been reported with currently available systemic therapies. IFN-α has been tested in both clinic and animal models and only moderate benefits have been observed. In animal models, similar modest antitumor efficacy has also been reported for IFN-λ, a new type of IFN that acts through its own receptor complex. In the present study, the antitumor efficacy of the combination of IFN-α and IFN-λ was tested in the BNL mouse hepatoma model. This study was accomplished by using either engineered tumor cells (IFN-α/IFN-λ gene therapy) or by directly injecting tumor-bearing mice with IFN-α/IFN-λ. Both approaches demonstrated that IFN-α/IFN-λ combination therapy was more efficacious than IFN monotherapy based on either IFN-α or IFN-λ. In complement to tumor surgery, IFN-α/IFN-λ combination induced complete tumor remission. Highest antitumor efficacy has been obtained following local administration of IFN-α/IFN-λ combination at the tumor site that was associated with strong NK cells tumor infiltration. This supports the use of IFN-α/IFN-λ combination as a new cancer immunotherapy for stimulating antitumor response after cancer surgery.

## INTRODUCTION

Hepatocellular carcinoma (HCC) is a major worldwide cancer with more than 660 thousand people diagnosed each year [1]. Potentially curative treatments like surgical resection or liver transplantation are possible, however tumor recurrence and metastasis frequently occur after resection and limit the overall survival [2, 3]. Postoperative IFN-α therapy appears to decrease recurrence after ablative therapies such as radiofrequency ablation (RFA) of HCV-related HCC [4]. Many studies have documented that IFN significantly suppresses the onset of HCC in patients with chronic hepatitis or liver cirrhosis. Thus, IFN-α therapy for HBV and HCV infections suppresses carcinogenesis and improves liver function [5, 6]. Moreover, IFN therapy eliminates HCV mRNA, and reduces the onset of HCC in patients with normalized transaminase levels [7]. Although IFN-α therapy suppressed HCC, complete tumor eradication is rarely obtained. Currently, tremendous efforts are made to improve IFN therapy for efficiently treating HCC and eliminating tumor recurrence which remains the main concern following liver resection [5, 6, 8]. In agreement with clinical studies, we have confirmed in BNL hepatoma model that IFN-α displayed potent antitumor activity without inducing a complete tumor remission [9]. Similar efficacy was obtained with IFN-λ. IFN-λ induces high NK cell activation and in contrast to IFN-α, it does not cause alteration of T regulatory (Treg) cells [9]. Since the antitumor mechanisms of IFN-α and IFN-λ were quite distinct, we hypothesized that combined action of IFN-α and IFN-λ would be more effective than IFN monotherapy. For this reason, we analyzed the efficacy of IFN-α/λ combination in cancer therapy.

Currently, IFN is administrated systemically to patients and often resulted in low antitumor efficacy and significant side effects due to out of targeting [10, 11]. For this reason, we explored the role of IFN in the tumor microenvironment. In the present study, we found that local administration of IFN at the tumor site in BNL hepatoma model, significantly improved IFN therapy. We have obtained a complete tumor remission when both IFN-α and IFN-λ were administrated to mice via gene therapy approach or by local IFN injection to the tumor site following tumor surgery. We demonstrated that this high antitumor efficacy of IFN-α/IFN-λ combination is associated with enhanced NK cell tumor infiltration and increased NK cell tumoricidal activity. Therefore our findings strongly suggest the use of IFN-α/IFN-λ combination as a novel therapeutic approach for treating HCC and eradicating tumor recurrence.

## RESULTS

### Effect of the IFN-α and IFN-λ combination treatment in the BNL hepatoma model

Our main goal was to evaluate the antitumor efficacy of IFN-α/IFN-λ combination and to address the role of IFN when locally introduced in the tumor microenvironment. We used a classical gene therapy approach to locally deliver the IFN at the tumor site and also tested direct IFN injection in the tumor area.

**Figure 1:**
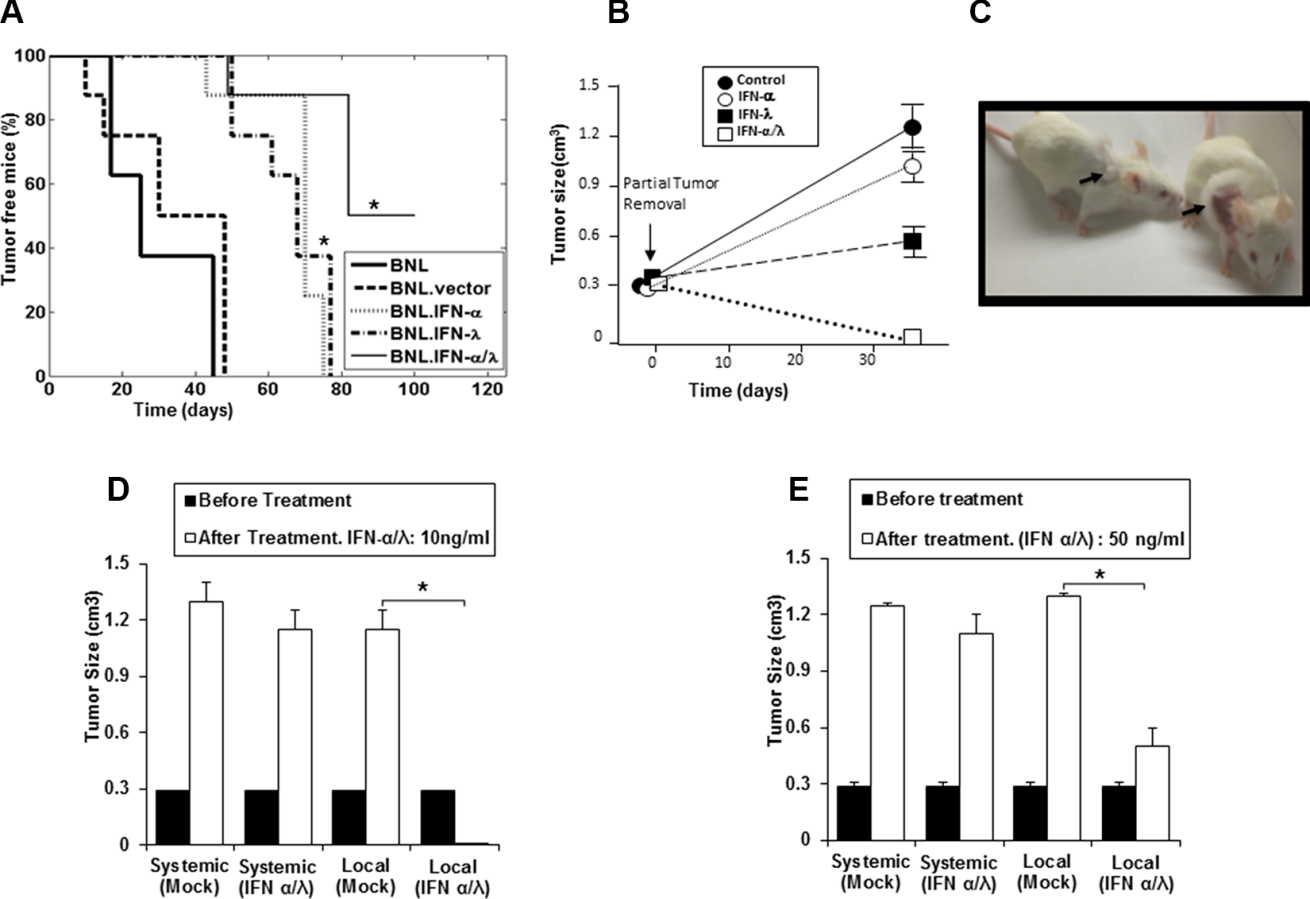
Synergisitic effects of IFN-α and IFN-λ on *in vivo* BNL tumor growth (**A**) Syngeneic BALB/c mice (*n* = 8) were injected s.c. in the flank with 10^6^ BNL, BNL.vector, BNL.IFN-λ, BNL.IFN-α cells, or a 50:50 combination of both IFN-producing cells. Data in the Kaplan-Meier survival curves are shown as percentage of tumor free mice. BNL.IFN-α or BNL.IFN-λ versus BNL.IFN-α/λ cells (*) *p* < 0.05 (one-way ANOVA). (**B**) Mice (*n* = 5 per group) were inoculated with 10^6^ parental BNL cells at the back. After tumor growth (1 cm^3^), partial tumor removal was performed (0.3 cm^3^ left). Mice were then treated with 10 ng of either IFN-α or IFN-λ alone, or a combination of IFN-α and IFN-λ (50% of each dose) at the tumor surgery site, starting two days post-surgery, three days a week for two weeks; and tumor growth was monitored. Data are presented as the mean tumor volume ± SE (*n* = 5). (**C**) Representative example showing a mouse, treated with IFN-α and IFN-λ combination (Left) and control (Right). Arrows indicate the tumor site and area of treatment with IFN-α and IFN-λ combination or control (Mock). (**D** and **E**) Mice (*n* = 5 per group) were inoculated with 10^6^ parental BNL cells at the neck. When the tumor reached around 1 cm^3^, partial tumor removal was performed. Mice were then treated locally at the tumor inoculation site at the neck or systemically (i. p) with either 10 ng (d) or 50 ng (e) of a combination of IFN-α and IFN-λ (50% of each IFN dose), three days a week for two weeks and starting two days post-surgery. Experiments are repeated 3 times and data presented as the mean tumor volume ± SE. (*) *p* < 0.05. *p* value determined by Mann-Whitney *U* test.

Using a gene therapy approach, we previously demonstrated that IFN-λ and IFN-α display antitumor activities in a HCC murine model by delaying the *in vivo* tumor growth of BNL hepatoma cells [9]. However, neither IFN-λ nor IFN-α completely suppressed tumor growth; only a delay in the onset of tumor development and slower tumor growth were observed. In the present studies, we determined whether the combination of IFN-λ and IFN-α was more effective in inhibiting tumor development than either agent alone. All mice injected with parental BNL cells, BNL.vector cells and BNL.IFN-λ or BNL.IFN-α cells developed tumors (Figure 1A). However, a delay in tumor growth was observed in mice injected with either BNL.IFN-α or BNL.IFN-λ cells; nevertheless tumors appeared in 100% of mice (Figure 1A). In contrast, only 50% of mice developed tumors after injection with a combination of BNL.IFN-α and BNL.IFN-λ (BNL.IFN-α/λ) cells, suggesting a concerted local action of IFN-α and IFN-λ in tumor eradication.

To address the potential benefit of IFN-α/λ combination as an effective therapy for cancer recurrence, mice were treated with IFN after partial tumor removal. Cancer recurrence is highly relevant in clinic and new therapies are needed. Even when the tumors are supposedly removed or the diseased organ replaced, residual cancer cells cause cancer recurrence. After cancer surgery (partial tumor removal), mice were subjected to IFN-α/λ combination therapy. As shown in Figure 1B and illustrated in Figure 1C, mice treated locally at the tumor site with the combination of IFN-α and IFN-λ demonstrated complete tumor remission, whereas injection of either IFN-α or IFN-λ alone had a modest tumor repressive effect.

The positive antitumor results obtained with the combination of IFN-α and IFN-λ prompted us to compare the effects of local, at the tumor site, vs. systemic intraperitoneal injections of the IFN-α/λ combination to tumor bearing mice after partial tumor removal. In contrast to the systemic administration of the IFNs (Figure 1D and 1E), local administration was found to be highly effective in mice after partial tumor removal, using two doses of the IFN combination, a lower dose (10 ng) and a higher dose (50 ng) (Figure 1D and 1E). At the high dose, local IFN administration induced marked tumor suppression (Figure 1E).However, higher IFN dosage was less efficacious than lower IFN dosage when used as local tumor treatment. The lower dose of the combined IFNs administered locally induced complete tumor remission (Figure 1C and 1D). This indicates that the presence of low concentration of both IFN-α and IFN-λ in the tumor microenvironment are particularly effective in eradicating tumors, remaining after resection. Therefore, local administration of low dose of IFN-α/λ to tumor site could be highly beneficial in clinic for the treatment of primary tumors and the prevention of tumor recurrence.

### Antitumor immunity induced by the combined IFN-α/λ treatment

**Figure 2:**
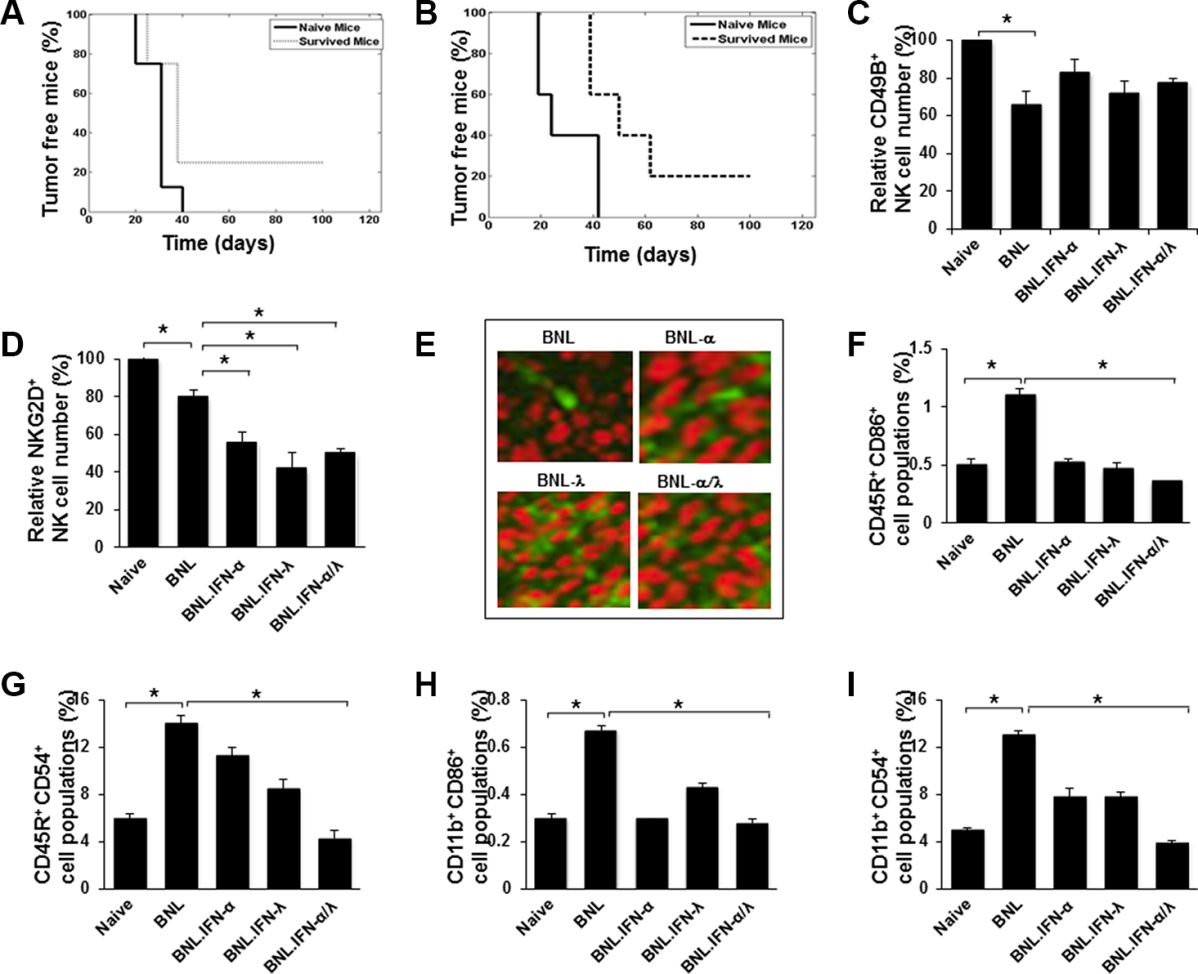
Antitumor immunity induced by the IFN-α/λ combination (**A** and **B**) Survived mice in the experiments represented in Figure 1A (*n* = 4) and Figure 1C (*n* = 5), respectively, were re-challenged with a s.c. injection at the original tumor inoculation site (left flank) with 10^6^ parental BNL cells. Mice were followed for 3 months and percentage of tumor free mice was determined. (**C** and **D**.) Blood was isolated from naive mice or mice at 15 days post injection with 10^6^ of parental BNL or engineered BNL cells, BNL.IFN-α, BNL.IFN-λ and the combination of BNL.IFN-α and BNL.IFN-λ (BNL.IFN-α/λ) cells (50% of each). The number of circulatory CD49B^+^ NK cells (c), CD49B^+^/NKG2D^+^ NK cells (d) was determined by flow cytometry in each group of mice. Relative cell numbers (%) are shown as the mean ± SE (*n* = 3 mice per data point). Relative cell numbers (%) are shown as the mean ± SE (*n* = 3 tumors per data point). (**E**) Immunohistochemical staining with green fluorescence of NK cells (CD49B+), infiltrating parental or engineered BNL hepatoma tumors. Images were taken on confocal fluorescence microscope (x250). (**F**–**I**) Assessment of circulatory CD45R+/CD86+ (f), CD45R+/CD54+ (g), CD11b+/CD86+ (h) and CD11b+/CD54+ (i) populations in mice blood. Blood was isolated from naive mice or mice at 15 days post injection with 10^6^ of parental BNL or engineered BNL cells, BNL.IFN-α, BNL.IFN-λ and the combination of BNL.IFN-α and BNL.IFN-λ (BNL.IFN-α/λ) cells (50% of each). The number of cells was determined in each group of mice and presented as relative cell numbers (%) and shown as the mean ± SE (*n* = 3 mice per data point). *P* values are calculated using Mann-Whitney *U* test. (*) *p* < 0.05.

In order to determine whether the mice that survived the tumor challenge following combined IFN treatment had generated long-lasting immunity, the mice were rechallenged with parental BNL cells. After 3 months, the majority (75–80%) of the mice developed tumors. Only 25% and 20% of the rechallenged mice, originating respectively from gene therapy and partial tumor removal approaches, were tumor free (Figure 2A and 2B), suggesting a lack of memory response despite a potential initial T cell response. Since NK cells have been implicated in the antitumor activity of IFN-α and IFN-λ [9, 14], we particularly focused our investigation on the role of NK cells on the marked antitumor activity of IFN-α/λ combination. We first analyzed NK cell number and activity in the blood of mice. As shown in Figure 2C, a marked reduction in circulatory NK cells was observed in mice challenged with tumor cells relative to naïve mice. Similarly, the number of circulatory NK cells was still reduced in mice bearing tumors expressing each type of IFN alone or in combination. Furthermore, the effect of IFN was associated with a decreased activation of NK cells in the peripheral blood as assessed by NKG2D expression (Figure 2D), suggesting that antitumor activity of IFNs did not correlate with activation status of systemic NK cells. However, in accordance with the high antitumor impact of IFNs delivered into the tumor (Figure 1B and 1C), local recruitment of NK cells could be promoted by IFNs. Indeed, tumor-immunohistology analysis showed high infiltration of NK cells, particularly in the presence of IFN-λ (Figure 2E). The fact that tumor infiltration of NK cells was reduced in mice injected with the combination of BNL.IFN-α/λ cells in comparison with BNL.IFN-λ could suggest a reduction of IFN-λ production by tumors that developed in mice. To test this hypothesis, we analyzed tumor samples *ex-vivo* for IFN production and indeed observed a significant reduction in IFN-λ secretion (data not shown), suggesting that in the context of the mixed BNL.IFN-α/λ cells, the growth of BNL.IFN-λ cells within the tumor was suppressed. Therefore, the IFN-α/λ combination might act locally to increase NK cell tumor targeting.

We next examined whether the antitumor activity of the IFN-α/λ combination is associated with a systemic decrease in the blood of other cell populations as demonstrated for NK cells. In comparison with naïve mice, we observed significant increases in B (CD45R+; Figure 2F and 2G) and macrophage/dendritic (CD11b+; Figure 2H and 2I) cell populations positive for the expression of CD86 and CD54 activation markers in the blood of mice injected with parental BNL cells. In contrast to parental BNL cells, engineered BNL cells did not induce significant changes in these cell populations, compared to naïve controls. A more significant decrease of either CD86+ or CD54+ cell population, reaching normal levels was detected in mice subjected to the BNL.IFN-α/λ cell injection, suggesting a link between the antitumor efficacy of IFN-α/λ combination and the reduction of the number of activated peripheral blood cells in tumor-bearing mice.

### Sensitization of BNL tumor cells to NK cell killing by the IFN-α/λ combination

**Figure 3:**
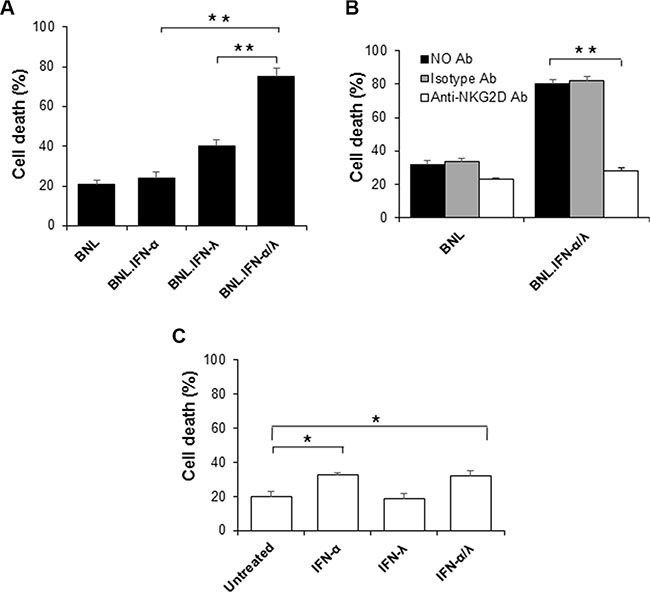
NK cells induced tumor immunotoxicity (**A**) NK cells were evaluated for their tumor toxicity on target cells, parental BNL or engineered BNL cells, BNL.IFN-α, BNL.IFN-λ and BNL.IFN-α/λ cells. After co-culture of NK cells (purified from spleen of naïve mice) with target cells, cells were harvested, PI stained and the amount of cell death was assessed by FACS as indicated in Supplementary Figure 1. Dead cells (%) are shown as mean ± SE (*n* = 3 by data points). (**B**) Effect of the blocking anti-NKG2D antibody (C7) on the promotion of tumor toxicity using IFN-α/λ-secreting BNL cells. After one hour incubation of anti-NKG2D or isotype control antibody with NK cells, tumor cytotoxicity was evaluated. (**C**) Effect of pretreatment of NK cells with IFN on the induction of NK cells cytotoxicity on parental BNL tumors. Dead cells (%) are shown as mean ± SE (*n* = 3 by data points, (**) *p* < 0.01, (*) *p* < 0.05). *P* values are calculated using Mann-Whitney *U* test.

**Figure 4:**
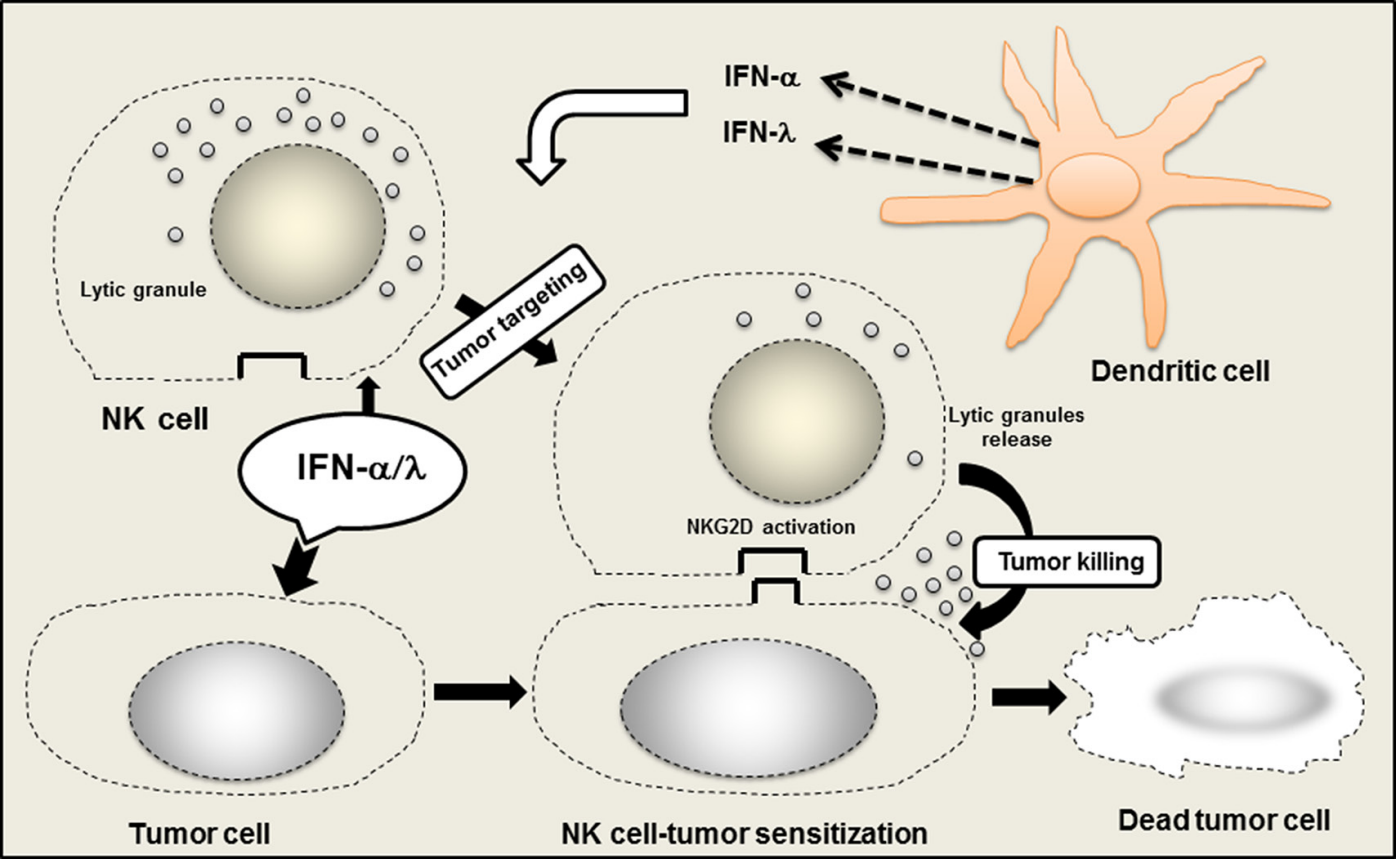
Role of NK cells in the promotion of anticancer efficacy of IFN-α/λ combination Within the tumor site, IFN-α/λ induced NK cells activation, mostly via tumor cell sensitization to NK cells. Concerted action of IFN-α and IFN-λ promotes NK cell tumor targeting and induces tumor eradication. IFN-α/λ is released in the tumor site by direct injection or through IFN secretion by dendritic cells (DCs) either conventional or plasmacytoid DCs. Activation of NKG2D system by IFN-α/λ appears crucial within the tumor microenvironment for the promotion of local NK cell tumor surveillance.

IFN-α/λ combination therapy induced marked antitumor activity. Apparently this antitumor effect was dependent on the concerted action of IFN-α and IFN-λ within the tumor microenvironment. Our tumor immunohistology analysis strongly suggested the involvement of NK cells, in agreement with previous reports, demonstrating that both IFN-α and IFN-λ promoted NK cell antitumor activities [9, 15, 16]. To address the role of NK cells in the antitumor activity induced by IFN-α/λ combination treatment, we have evaluated NK cell-mediated tumor cytotoxicity. Purified NK cells from spleens of naïve mice were tested for their tumor cytotoxicity on parental BNL cells and engineered BNL cells constitutively producing IFN-α and IFN-λ. As shown in Figure 3A, the strongest cell killing (tumor cytotoxicity) was observed in BNL cells producing both IFN-α and IFN-λ. Therefore concerted action of IFN-α and IFN-λ is critical in tumor cells sensitization to NK cell-mediated tumor cytotoxicity. To address whether NK cell-mediated tumor cytotoxicity involved direct interaction between NK cells and BNL tumor cells, we evaluated the involvement of NKG2D system, known to play a central role in inducing NK cell tumoricidal functions. To test this, naïve NK cells were pre-incubated with a blocking anti-NKG2D antibody and NK cells-mediated tumor cytotoxicity was evaluated using parental and IFN-α/λ-secreting BNL cells. As shown in Figure 3B, significant reduction of cell death was observed in IFN-α/λ-secreting BNL cells, strongly suggesting that NKG2D engagement was necessary for NK cell-mediated cytotoxicity against those tumor cells. However pre-exposure of NK cells to IFN-α/λ moderately affected their cytotoxic activity on parental BNL cells (Figure 3C). Therefore, the antitumor effects resulting from IFN-α/λ appear to depend mainly on sensitization of BNL cells to NK cell targeting and lysis. Therefore, the presence of both IFN-α and IFN-λ in the tumor microenvironment appear crucial in tumor sensitization to NK cell mediated antitumor activity and clearance of the host from cancer (Figure 4).

## DISCUSSION

Although, previous studies demonstrated that either IFN-α or IFN-λ alone induces significant tumor suppression in the BNL hepatoma model, only a delay in tumor growth was observed with no increase in survival [9]. In the present study, we evaluated the combination of IFN-α and IFN-λ as a potential new antitumor therapy. Remarkably, the combination of IFN-α and IFN-λ was highly effective in suppressing tumor growth in mice. This was observed by using either a gene therapy approach (Figure 1A), or by directly injecting tumor-bearing mice with IFN-α and IFN-λ (Figure 1B and 1C). In accordance with IFN adjuvant therapy, which mostly used in clinic after tumor surgery [10], combination of IFN-α and IFN-λ was more successful than IFN monotherapy in eradicating the tumor and preventing its recurrence (Figure 1B and 1C). In contrast to systemic administration, local administration of IFN-α and IFN-λ was more efficient in tumor elimination, particularly when lower IFN doses were administrated (Figure 1C and 1D). Higher amounts of IFN-α and IFN-λ were less efficient in suppressing tumor growth. Because low doses of IFN-α and IFN-λ were highly effective in suppressing tumor growth, limited adverse effects associated with IFN-α/λ combination therapy could be expected in patients. Presence of suitable level of IFN-α and IFN-λ in the tumor microenvironment seems necessary for optimal tumor eradication. In clinic, significant lymphotoxicity has been associated with high doses of IFN-α, suggesting that excessive IFN dosage may jeopardize the beneficial immune response against tumor cells. [10, 11]. Our findings show that concerted action of IFN-α and IFN-λ is crucial in promoting a strong NK cell-antitumor immunity, mostly occurring via tumor sensitization to NK cells (Figure 3A) rather than a direct modulation of NK cell functions (Figure 3D). In contrast to IFN-α, we did not observe any direct response of NK cells to IFN-λ (Figure 3D), in agreement with our previous report [9] and other studies, demonstrating that IFN-λ was not acting directly on NK cells [17, 18]. Therefore, it is likely that activation of NK cells is promoted to higher extent by the direct action of IFN-α, whereas simultaneous action of IFN-λ and IFN-α on tumor cells makes them a better target for NK cell-mediated cytotoxicity. We have demonstrated that NK cell tumor targeting occurred via the activator receptor NKG2D [19–21]. Upregulation of NKG2D ligand, H60 by IFN-λ has been suggested in B16 melanoma model [22]. However a concerted action of IFN-α and IFN-λ on tumor cells may have a greater impact on the expression balance of NK cell activator and inhibitor receptors that control NK cell tumoricidal functions [20, 21].

Our study supports the use of adoptive transfer of NK cells as therapeutic strategy for HCC treatment in agreement with the recent reports [23, 24]. However, it suggests that alternative strategies, not based on the direct activation of NK cells as commonly reported can be also used to promote NK cell therapy [25]. For establishing a successful NK cell therapy against HCC and probably other cancers, our findings show that tumor cell sensitization to NK cell-mediated tumor cytotoxicity is crucial. Administration of IFN-α and IFN-λ in the tumor microenvironment would be central in promoting tumor sensitization to NK cells. Appropriate production of IFN-α and IFN-λ in the tumor microenvironment may occur naturally via potential tumor sensoring pathways such STING [26, 27]. Deficiency on those pathways may impair IFN production and enhance tumor development. Introduction of IFN-α and IFN-λ in the tumor microenvironment may be beneficial for rectifying potential deficiencies of tumor sensoring pathways and led to the induction of antitumor responses.

It is also noteworthy to mention that recent studies uncovered association between a set of linked polymorphisms within the IFN-λ locus and the success of IFN-α-based treatment in patients chronically infected with HCV [28]. These polymorphisms may modulate expression or activity of IFN-λ [29, 30] or IFN-λR1 [31] and, in turn, affect IFN-α therapy. The IFN-λ polymorphism was reported to be also associated with altered functions of NK cells [32, 33]. In addition, treatment of cells with IFN-λ induces several negative regulators of IFN signaling and desensitizes cells to IFN-α [34–36]. All these studies suggest the existence of a multi-leveled crosstalk between IFN-λ and IFN-α, which underlies synergistic antitumor activities of these IFNs observed in the current study.

In conclusion, our study demonstrates that IFN-α and IFN-λ possess non-redundant antitumor activities and emphasizes a concerted role of IFN-α and IFN-λ in promoting NK cell tumor targeting. Additive and complementary antitumor mechanisms of type I and type III IFNs observed in the current and previous studies [9, 15, 37, 38] suggest that the combinatorial therapy using IFN-α and IFN-λ should be further explored to improve IFN-based cancer immunotherapy.

## MATERIALS AND METHODS

### Cells and transfections and expression plasmids

BALB/c-derived murine hepatocellular carcinoma BNL cells were maintained in DMEM medium with 10% FBS. Expression plasmids pEF-mIFN-λ2 and pEF-mIFN-α7 were described previously [9, 12]. pEF-mIFN-λ2 and pEF-mIFN-α7 plasmids were stably transfected into 10^6^ BNL cells using transit-LT1 reagent (Mirus, Madison, WI). G418-resistant cells were selected by using 500 μg/ml of Geneticin (Invitrogen, Carlsbad, CA) as previously described [9, 12].

### Flow cytometric analysis

To assess the effect of IFN-α/IFN-λ combination on the activation of peripheral blood mononuclear cell (PBMC) populations in mice harboring tumors, whole blood was extracted from mice (after anesthesia) 14 days following tumor injection with parental or BNL cells, expressing IFN-α and IFN-λ. Blood was collected in tubes containing citrate sodium to prevent coagulation. Before flow cytometry analysis, blood was cleared from red blood cells by using lysis buffer as recommended by the manufacturer (Sigma, Saint Louis MO, USA). Similar volume of PBMCs (100 μl) was used. To determine the absolute number of cells in each sample, we used count bright beads before flow cytometry acquisition. Blood from each group (3–5 mice) was individually analyzed and % of total or relative cell populations was determined.

Activated PBMCs, B cells (CD45R+/CD86+, CD45R+/CD54+), monocytes (CD11b+/CD86+, CD11b+/CD54+) and NK cells (CD49B+/NKG2D+) were double stained with rat monoclonal antibodies against CD45R APC, CD11b PE (CALTAG Laboratories, Burlingame, CA) or CD49B FITC (Pan NK cells) (BioLegend, San Diego, CA, USA), along with CD54 FITC, CD86 FITC and NKG2D PE (BioLegend, San Diego, CA, USA). Stained cells (10^4^) were analyzed by flow cytometry as previously described [9, 12] using Beckman Coulter Gallios Flow Cytometry (compensation was performed by Gallios software during sample acquisition).

### Tumor transplantation and IFN treatment

Immunocompetent female BALB/c mice (6–8 weeks old) were obtained from Jackson Laboratory (Bar Harbor, ME, USA) and maintained in a pathogen-free barrier facility. BALB/c mice were injected subcutaneously (s.c) into flank or back with 10^6^ BNL cells (parental or transfected with empty plasmid), BNL.IFN-α cells, BNL.IFN-λ cells or a 50:50 combination of BNL.IFN-α and BNL.IFN-λ cells in 0.1 ml of PBS. Tumor growth was evaluated by palpation of the injection site every 2 days. After mice developed tumors (~ 0.5 cm^3^), a lower dose (10 ng) or higher dose (50 ng) of IFN-α or IFN-λ alone, or in combination (50% of each), diluted in 0.1 ml of PBS was injected locally at the tumor site or intraperitoneally every 3 days for 2 weeks. Mice were followed for tumor development for additional 3 weeks. In other groups, similar doses of IFN were administrated to mice after partial surgical tumor removal (~0.3 cm^3^ remaining tumor from ~1 cm^3^ tumor). IFN treatment was started 2 days after the surgery and maintained every 3 days for 2 weeks. Tumor volume was measured by a Vernier caliper. Tumor volume (cubic millimeter) was calculated using the formula ab^2^/2, where a = largest diameter and b = smallest diameter. Animals with excessive tumor burden (≥ 1 cm^3^) were euthanized. Mice that rejected tumors were re-challenged with 10^6^ parental BNL cells and followed for potential tumor redevelopment for 3 months.

### NK cell cytotoxicity, immunohistochemistry and NKG2D blocking

NK cell cytotoxicity was assessed by FACS and Propidium Iodide (PI) staining. 5 × 10^3^ target cells (Parental BNL cells and BNL cells expressing IFN-α and/or IFN-λ) and 2.5 × 10^4^ NK cells were co-incubated for 4 hours at 37°C and 5% CO_2_. NK cells were pretreated or not for 12 hours with IFN prior immunotoxicity assay [10 ng of IFN-α or IFN-λ alone, or in combination (50% of each)], The cells were harvested and resuspended in 500 μl of PBS. 5 μl of PI was added to the cells and cell viability/mortality was analyzed by FACS (forward and scatter gated) as illustrated in Supplementary Figure 1.

Immunohistochemistry of tumors for NK cell infiltration were performed as previously described [9, 13]. For NKG2D blocking, we used the C7 monoclonal antibody which reacts with the mouse NKG2D (eBioscience, San Diego CA, USA). Armenian hamster IgG isotype control (clone: eBio299Arm, eBioscience, San Diego CA, USA) was included. 2.5 × 10^4^ NK cells were pre-incubated with the anti-NKG2D blocking antibody or isotype control for 1 hour (37°C 5% CO_2_) at 20 μg/ml prior to immunotoxicity assay. Immunotoxicity assay with NK cells was preformed similarly as above by co-incubating 5 × 10^3^ target cells (parental BNL cells and BNL cells expressing IFN-α and/or IFN-λ) with 2.5 × 10^4^ NK cells. Prior to immunotoxicity assay, NK cells were treated or not with IFN-α/λ (10 ng/ml of IFN-α or IFN-λ for 2–10 hours). Isolation of NK cells from single cell suspensions of murine spleen was performed using the NK cell isolation kit II (Miltenyi Biotech, Auburn CA, USA).

### Data analysis

The Kaplan-Meier estimator was used to calculate the median survival (tumor appearance) time and to derive tumor appearance (survival) curves. Statistical analysis was performed using a one-way ANOVA and Mann-Whitney *U* test. Differences were considered statistically significant at *p* < 0.05.

## SUPPLEMENTARY MATERIALS FIGURE



## References

[1] Sherman M (2010). Hepatocellular carcinoma: epidemiology, surveillance, and diagnosis. Seminars in liver disease.

[2] Georgiades C, Geschwind JF, Harrison N, Hines-Peralta A, Liapi E, Hong K, Wu Z, Kamel I, Frangakis C (2012). Lack of Response after Initial Chemoembolization for Hepatocellular Carcinoma: Does It Predict Failure of Subsequent Treatment?. Radiology.

[3] Melloul E, Lesurtel M, Carr BI, Clavien PA (2012). Developments in liver transplantation for hepatocellular carcinoma. Seminars in oncology.

[4] Shimomura S, Ikeda N, Saito M, Ishii A, Takashima T, Sakai Y, Yoshikawa S, Aizawa N, Tanaka H, Iwata Y, Enomoto H, Imanishi H, Yamamoto T (2010). Long-term interferon therapy after radiofrequency ablation is effective in treating patients with HCV-associated hepatocellular carcinoma. Hepatol Int.

[5] Lin SM, Yu ML, Lee CM, Chien RN, Sheen IS, Chu CM, Liaw YF (2007). Interferon therapy in HBeAg positive chronic hepatitis reduces progression to cirrhosis and hepatocellular carcinoma. J Hepatol.

[6] Yu ML, Lin SM, Chuang WL, Dai CY, Wang JH, Lu SN, Sheen IS, Chang WY, Lee CM, Liaw YF (2006). A sustained virological response to interferon or interferon/ribavirin reduces hepatocellular carcinoma and improves survival in chronic hepatitis C: a nationwide, multicentre study in Taiwan. Antivir Ther.

[7] Ishikawa T (2008). Secondary prevention of recurrence by interferon therapy after ablation therapy for hepatocellular carcinoma in chronic hepatitis C patients. World journal of gastroenterology.

[8] Ishikawa T, Higuchi K, Kubota T, Seki K, Honma T, Yoshida T, Kamimura T (2012). Combination PEG-IFN a-2b/Ribavirin Therapy Following Treatment of Hepatitis C Virus-Associated Hepatocellular Carcinoma is Capable of Improving Hepatic Functional Reserve and Survival. Hepato-gastroenterology.

[9] Abushahba W, Balan M, Castaneda I, Yuan Y, Reuhl K, Raveche E, de la Torre A, Lasfar A, Kotenko SV (2010). Antitumor activity of type I and type III interferons in BNL hepatoma model. Cancer Immunol Immunother.

[10] Jonasch E, Haluska FG (2001). Interferon in oncological practice: review of interferon biology, clinical applications, and toxicities. The oncologist.

[11] Pestka S, Krause CD, Walter MR (2004). Interferons, interferon-like cytokines, and their receptors. Immunol Rev.

[12] Lasfar A, Lewis-Antes A, Smirnov SV, Anantha S, Abushahba W, Tian B, Reuhl K, Dickensheets H, Sheikh F, Donnelly RP, Raveche E, Kotenko SV (2006). Characterization of the mouse IFN-lambda ligand-receptor system: IFN-lambdas exhibit antitumor activity against B16 melanoma. Cancer research.

[13] Willemen Y, Van den Bergh JM, Lion E, Anguille S, Roelandts VA, Van Acker HH, Heynderickx SD, Stein BM, Peeters M, Figdor CG, Van Tendeloo VF, de Vries IJ, Adema GJ (2015). Engineering monocyte-derived dendritic cells to secrete interferon-alpha enhances their ability to promote adaptive and innate anti-tumor immune effector functions. Cancer Immunol Immunother.

[14] Sato A, Ohtsuki M, Hata M, Kobayashi E, Murakami T (2006). Antitumor activity of IFN-lambda in murine tumor models. J Immunol.

[15] Souza-Fonseca-Guimaraes F, Young A, Mittal D, Martinet L, Bruedigam C, Takeda K, Andoniou CE, Degli-Esposti MA, Hill GR, Smyth MJ (2015). NK cells require IL-28R for optimal *in vivo* activity. Proc Natl Acad Sci U S A.

[16] Zitvogel L, Galluzzi L, Kepp O, Smyth MJ, Kroemer G (2015). Type I interferons in anticancer immunity. Nat Rev Immunol.

[17] de Groen RA, Boltjes A, Hou J, Liu BS, McPhee F, Friborg J, Janssen HL, Boonstra A (2015). IFN-lambda-mediated IL-12 production in macrophages induces IFN-gamma production in human NK cells. Eur J Immunol.

[18] Morrison MH, Keane C, Quinn LM, Kelly A, O'Farrelly C, Bergin C, Gardiner CM (2014). IFNL cytokines do not modulate human or murine NK cell functions. Hum Immunol.

[19] Langers I, Renoux VM, Thiry M, Delvenne P, Jacobs N (2012). Natural killer cells: role in local tumor growth and metastasis. Biologics: targets & therapy.

[20] Lanier LL (2008). Up on the tightrope: natural killer cell activation and inhibition. Nat Immunol.

[21] Vivier E, Raulet DH, Moretta A, Caligiuri MA, Zitvogel L, Lanier LL, Yokoyama WM, Ugolini S (2011). Innate or adaptive immunity? The example of natural killer cells. Science.

[22] Wongthida P, Diaz RM, Galivo F, Kottke T, Thompson J, Pulido J, Pavelko K, Pease L, Melcher A, Vile R (2010). Type III IFN interleukin-28 mediates the antitumor efficacy of oncolytic virus VSV in immune-competent mouse models of cancer. Cancer research.

[23] Nishida S, Levi DM, Tzakis AG (2013). Liver natural killer cell inoculum for liver transplantation with hepatocellular carcinoma. Curr Opin Organ Transplant.

[24] Ohira M, Nishida S, Tryphonopoulos P, Tekin A, Selvaggi G, Moon J, Levi D, Ricordi C, Ishiyama K, Tanaka Y, Ohdan H, Tzakis AG (2012). Clinical-scale isolation of interleukin-2-stimulated liver natural killer cells for treatment of liver transplantation with hepatocellular carcinoma. Cell Transplant.

[25] Childs RW, Carlsten M (2015). Therapeutic approaches to enhance natural killer cell cytotoxicity against cancer: the force awakens. Nat Rev Drug Discov.

[26] Ahn J, Xia T, Konno H, Konno K, Ruiz P, Barber GN (2014). Inflammation-driven carcinogenesis is mediated through STING. Nat Commun.

[27] Woo SR, Fuertes MB, Corrales L, Spranger S, Furdyna MJ, Leung MY, Duggan R, Wang Y, Barber GN, Fitzgerald KA, Alegre ML, Gajewski TF (2014). STING-dependent cytosolic DNA sensing mediates innate immune recognition of immunogenic tumors. Immunity.

[28] Suppiah V, Moldovan M, Ahlenstiel G, Berg T, Weltman M, Abate ML, Bassendine M, Spengler U, Dore GJ, Powell E, Riordan S, Sheridan D, Smedile A (2009). IL28B is associated with response to chronic hepatitis C interferon-alpha and ribavirin therapy. Nat Genet.

[29] McFarland AP, Horner SM, Jarret A, Joslyn RC, Bindewald E, Shapiro BA, Delker DA, Hagedorn CH, Carrington M, Gale M, Savan R (2014). The favorable IFNL3 genotype escapes mRNA decay mediated by AU-rich elements and hepatitis C virus-induced microRNAs. Nat Immunol.

[30] Terczynska-Dyla E, Bibert S, Duong FH, Krol I, Jorgensen S, Collinet E, Kutalik Z, Aubert V, Cerny A, Kaiser L, Malinverni R, Mangia A, Moradpour D (2014). Reduced IFNlambda4 activity is associated with improved HCV clearance and reduced expression of interferon-stimulated genes. Nat Commun.

[31] Duong FH, Trincucci G, Boldanova T, Calabrese D, Campana B, Krol I, Durand SC, Heydmann L, Zeisel MB, Baumert TF, Heim MH (2014). IFN-lambda receptor 1 expression is induced in chronic hepatitis C and correlates with the IFN-lambda3 genotype and with nonresponsiveness to IFN-alpha therapies. J Exp Med.

[32] Golden-Mason L, Bambha KM, Cheng L, Howell CD, Taylor MW, Clark PJ, Afdhal N, Rosen HR (2011). Natural killer inhibitory receptor expression associated with treatment failure and interleukin-28B genotype in patients with chronic hepatitis C. Hepatology.

[33] Meng Q, Rani MR, Sugalski JM, Judge CJ, Phat S, Rodriguez B, Blanton RE, Anthony DD (2014). Natural cytotoxicity receptor-dependent natural killer cytolytic activity directed at hepatitis C Virus (HCV) is associated with liver inflammation, African American race, IL28B genotype, and response to pegylated interferon/ribavirin therapy in chronic HCV infection. J Infect Dis.

[34] Burkart C, Arimoto K, Tang T, Cong X, Xiao N, Liu YC, Kotenko SV, Ellies LG, Zhang DE (2013). Usp18 deficient mammary epithelial cells create an antitumour environment driven by hypersensitivity to IFN-lambda and elevated secretion of Cxcl10. EMBO Mol Med.

[35] Francois-Newton V, Magno de Freitas Almeida G, Payelle-Brogard B, Monneron D, Pichard-Garcia L, Piehler J, Pellegrini S, Uze G (2011). USP18-based negative feedback control is induced by type I and type III interferons and specifically inactivates interferon alpha response. PloS one.

[36] Makowska Z, Duong FH, Trincucci G, Tough DF Heim MH (2011). Interferon-beta and interferon-lambda signaling is not affected by interferon-induced refractoriness to interferon-alpha *in vivo*. Hepatology.

[37] Fujie H, Tanaka T, Tagawa M, Kaijun N, Watanabe M, Suzuki T, Nakayama K, Numasaki M (2011). Antitumor activity of type III interferon alone or in combination with type I interferon against human non-small cell lung cancer. Cancer Sci.

[38] Stiff A, Carson Iii W (2015). Investigations of interferon-lambda for the treatment of cancer. J Innate Immun.

