# Supplementary material for: Concerted action of IFN-α and IFN-λ induces local NK cell immunity and halts cancer growth
Source: Oncotarget. 2016 Jun 24;7(31):49259–67. doi: 10.18632/oncotarget.10272 (PMC5226505; doi:10.18632/oncotarget.10272)
Supplement: Supplementary file 1 [file oncotarget-07-49259-s001.pdf]

Concerted action of IFN-α and IFN-λ induces local NK cell immunity and halts cancer growth

Supplementary Materials

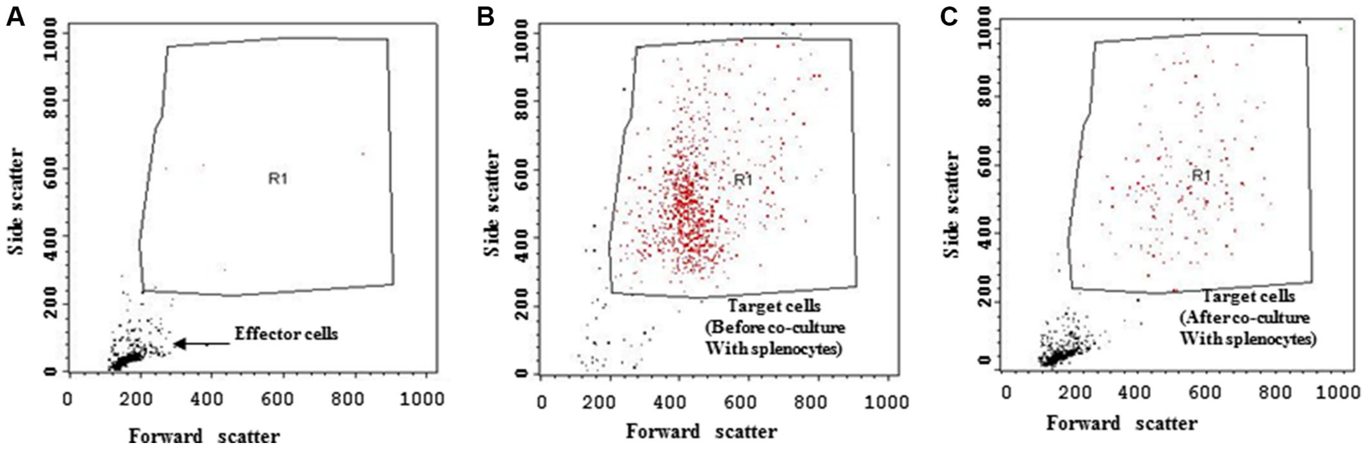

Supplementary Figure S1: Assessment of tumor cytotoxicity by using PI staining and FACS. (**A**) Slide scatter/Forward scatter gating of effector cells (splenocytes). (**B**) Slide scatter/Forward scatter gating of target cells (parental or engineered BNL cells). (**C**) Slide scatter/Forward scatter gating of co-cultured cells (effector and target cell).Prior FACS analysis, cell are stained with Propidium lodide (PI).
